# Effectiveness of Individualized Nursing in Perioperative Management of Patients With Obstructive Sleep Apnea: A Retrospective Cohort Study

**DOI:** 10.1155/carj/9293028

**Published:** 2026-07-02

**Authors:** Qunyu Lin, Yihong Yang, Chunmiao Huang, Dongdong Huang, Chao Fang

**Affiliations:** ^1^ Department of Otolaryngology-Head and Neck Surgery, The First Hospital of Putian, Putian, 351100, Fujian, China

**Keywords:** individualized nursing, obstructive sleep apnea, perioperative management, quality of life, respiratory safety

## Abstract

**Background:**

Patients with obstructive sleep apnea (OSA) are at increased risk of perioperative complications, such as hypoxemia, respiratory depression, and airway obstruction. This study aims to evaluate the effectiveness of individualized nursing in the perioperative management of patients with OSA.

**Methods:**

We retrospectively analyzed 107 patients with OSA undergoing elective surgery between January 2023 and January 2025, who were allocated to a conventional group (*n* = 55) or an individualized group (*n* = 52) according to the nursing approach. Perioperative respiratory safety (postanesthesia care unit [PACU], minimum oxygen saturation [SpO_2_], Aldrete score, and respiratory adverse events within 48 h), analgesia and functional recovery (visual analog scale [VAS] scores, opioid consumption, time to first ambulation), postoperative Epworth Sleepiness Scale (ESS) scores, continuous positive airway pressure (CPAP) adherence, Short Form‐36 (SF‐36) quality of life, complications, ICU transfer, length of stay, and hospitalization costs were compared between groups.

**Results:**

Compared with the conventional group, the individualized nursing group showed significantly higher PACU minimum SpO_2_ (*p* = 0.001) and Aldrete scores (*p* < 0.001), a lower incidence of postoperative respiratory adverse events within 48 h (*p* < 0.05), reduced 24‐h and 48‐h VAS scores and opioid consumption (*p* < 0.05), and earlier ambulation (*p* < 0.001). During the 6‐month follow‐up, the individualized group demonstrated lower ESS scores, improved CPAP adherence, and greater improvements across all SF‐36 domains (all *p* < 0.001), as well as reduced overall complications, ICU transfer rates, length of hospital stay, and hospitalization costs (*p* < 0.05).

**Conclusion:**

Individualized nursing is associated with improved perioperative respiratory safety and recovery quality, better long‐term sleep outcomes and quality of life, and reduced complications and healthcare resource use in patients with OSA.

## 1. Introduction

Obstructive sleep apnea (OSA) is a chronic disorder characterized by recurrent upper airway obstruction during sleep, often resulting in reduced or paused airflow and frequent arousals [1, 2]. Clinically, patients commonly present with nocturnal snoring, excessive daytime sleepiness, impaired attention, and reduced sleep quality, while bed partners often witness apneic episodes. Some patients also experience morning dry mouth, hypertension, and other related symptoms [1, 2]. The heterogeneity of OSA is reflected in its broad symptom spectrum and diverse phenotypes, with different patients exhibiting sleep disturbances, hypersomnolence, or subtypes with less pronounced classic symptoms [3].

Global epidemiological data indicate a marked increase in OSA prevalence, with the proportion of individuals at moderate to high risk steadily rising, partly driven by obesity and population aging [4, 5]. For instance, in Cyprus, the prevalence of moderate‐ to high‐risk OSA among adult men reaches 50%, compared with 18% in women. Similar studies from Asia and North Africa have demonstrated a positive correlation between OSA prevalence, body mass index (BMI), and age, with a continuous upward trend over recent years [4–7]. However, the majority of OSA cases remain undiagnosed, leading to a significant underestimation of the disease burden; actual prevalence is often substantially higher than reported [8, 9]. OSA not only disrupts sleep architecture but also substantially impairs daytime functioning and quality of life, increasing the risk of depression, anxiety, and cognitive dysfunction. It is also strongly associated with chronic conditions, such as cardiovascular and metabolic diseases, and diabetes [1, 2, 10]. Moreover, OSA imposes a considerable economic burden on patients, healthcare systems, and society, encompassing direct medical costs, sequelae‐related expenses, and indirect social costs, with annual societal losses estimated to far exceed direct healthcare expenditures [9, 11].

During the perioperative period, patients with OSA face specific risks, particularly in surgical settings, including airway complications, cardiopulmonary dysfunction, postoperative respiratory failure, arrhythmias, and thromboembolic events [12–14]. Due to heightened sensitivity to anesthetics and analgesics and increased risk of airway obstruction, perioperative management is considerably more complex, with potential challenges including difficult intubation, ventilation issues, and postoperative respiratory depression [12, 13]. Conventional nursing measures primarily involve preoperative screening, standard monitoring, early detection, and supportive management of complications. However, they remain insufficient in preventing adverse events and enhancing patient adherence and often fail to comprehensively address individual phenotypic variability and comorbidity profiles [15, 16]. The concept of individualized nursing emphasizes tailored management strategies based on patients’ anatomical characteristics, clinical phenotypes, and comorbidities, incorporating multidimensional interventions and active patient engagement. In theory, this approach can optimize safety, adherence, and effectiveness, potentially bridging gaps in conventional care [15–17].

This retrospective cohort study aims to systematically evaluate the effectiveness of individualized nursing in the perioperative management of patients with OSA. The findings are expected to provide evidence for optimizing nursing pathways, improving patient outcomes, and promoting innovation in perioperative care models.

## 2. Methods

### 2.1. Study Design and Participants

This study was a single‐center retrospective cohort study. We retrospectively collected perioperative clinical and nursing data of patients with OSA who underwent elective surgery at the Department of Otolaryngology‐Head and Neck Surgery, The First Hospital of Putian, between January 2023 and January 2025. Patients were assigned to either the conventional group (*n* = 55) or the individualized group (*n* = 52) according to the nursing approach documented in the electronic nursing records. Data were obtained from the hospital electronic medical record system, anesthesia information management system, nursing information system, and hospital billing database. The study protocol was approved by the Ethics Committee of the First Hospital of Putian (Approval No. 2026‐009). Given that this study involved retrospective analysis of anonymized data, the Ethics Committee waived the requirement for written informed consent. All patient identifiers were de‐identified after data extraction, and access was strictly restricted to ensure data security and privacy protection.

Inclusion criteria include the following: (1) age ≥ 18 years; (2) OSA confirmed by polysomnography (PSG); (3) undergoing elective surgery under general or regional anesthesia; (4) postoperative continuous monitoring in a general ward for ≥ 24 h; (5) complete perioperative nursing records, anesthesia records, and cost data; and (6) at least six months of follow‐up completed.

Exclusion criteria include the following: (1) central or mixed sleep apnea; (2) postoperative delayed extubation or requirement for reintubation; (3) severe comorbidities, such as New York Heart Association (NYHA) class IV heart failure or severe respiratory dysfunction with PaO_2_/FiO_2_ < 200 mmHg, as these patients typically require intensive multidisciplinary management that may substantially influence perioperative outcomes independent of nursing interventions; and (4) missing critical clinical data that could compromise statistical analysis.

### 2.2. Standard Care

Patients in the conventional group received perioperative nursing according to the hospital’s established routine protocols. Preoperatively, the responsible nurse provided general health education, including surgical precautions, fasting requirements, and routine postoperative care instructions, without OSA risk stratification or specialized sleep management. Intraoperatively, care followed standard nursing procedures in coordination with anesthesia and surgical teams, without implementation of specific airway‐optimizing positions or enhanced respiratory monitoring. Postoperatively, patients received intermittent SpO_2_ monitoring and conventional oxygen therapy in the general ward. Analgesia was administered according to departmental standard protocols, without individualized adjustments based on OSA risk. Nursing staff provided routine guidance for ambulation and diet but did not implement systematic respiratory training, sleep‐position interventions, or enhanced continuous positive airway pressure (CPAP) adherence management. No structured follow‐up program was arranged after discharge; patients received only general health guidance through standard outpatient visits.

### 2.3. Individualized Nursing

#### 2.3.1. Preoperative Phase

Specialized nurses conducted a systematic OSA risk assessment for each patient, including PSG parameters (apnea–hypopnea index (AHI), minimum oxygen saturation (SpO_2_), and nocturnal hypoxemia index), STOP‐Bang score, BMI, and comorbidities, followed by risk stratification and tailored management. Patients with prior CPAP use received reinforced guidance on nightly usage and adherence (≥ 4 h/night) to ensure uninterrupted preoperative utilization. Preoperative interventions also included upper airway patency training and health education, such as tongue‐base elevation, oropharyngeal muscle function exercises, and respiratory coordination training, along with guidance on sleep positioning (lateral or semi‐upright) to reduce supine‐related airway collapse. Individualized psychological interventions were provided for patients with anxiety or stress, including disease education, surgical process explanations, and relaxation training, aiming to enhance treatment compliance. Preoperative measures were further integrated with the anesthetic plan, encompassing bedtime medication, fasting management, and airway preparation to mitigate perioperative risk.

#### 2.3.2. Intraoperative Phase

The nursing team assisted anesthesiologists in optimizing patient positioning and airway management, applying head‐elevated or mild reverse Trendelenburg positions as needed to improve airway patency. Continuous monitoring of SpO_2_, respiratory parameters, and hemodynamic status was performed, with any abnormalities promptly communicated to the anesthesia team. Nurses also assisted in implementing multimodal analgesia strategies to minimize opioid use while maintaining temperature and fluid balance, ensuring physiological stability throughout the procedure.

#### 2.3.3. Postoperative Phase

Upon transfer to the general ward, continuous SpO_2_ monitoring was conducted for 12–24 h. CPAP or positional interventions were initiated promptly in response to hypoxemia (SpO_2_ < 90%), apnea episodes, or upper airway obstruction. Patients were guided through respiratory function exercises (deep breathing, pursed‐lip breathing, and thoracic mobility exercises) and encouraged to engage in early mobilization. Individualized analgesic strategies were applied to balance pain control with respiratory safety. During the postanesthesia care unit (PACU) stay, the nursing team recorded Aldrete scores to evaluate anesthetic recovery quality. Before discharge, structured health education was provided, covering CPAP usage, sleep positioning, weight management, smoking and alcohol cessation, and long‐term follow‐up care. Follow‐up assessments were conducted at 1, 3, and 6 months postoperatively via outpatient visits or telephone, evaluating ESS scores, nocturnal snoring and awakening episodes, CPAP adherence, and quality of life.

### 2.4. Outcome Measures

#### 2.4.1. Perioperative Respiratory Safety Outcomes

The primary outcomes were perioperative respiratory safety indicators, used to evaluate the impact of individualized nursing on respiratory stability in patients with OSA. Data were retrospectively collected from anesthesia, PACU, and ward records, including minimum oxygen saturation (SpO_2_) during PACU recovery, respiratory‐related adverse events within 48 h postoperatively, PACU recovery quality, and the need for respiratory support during hospitalization. The minimum peripheral SpO_2_ in the PACU reflects oxygenation during the postoperative awakening phase and is expressed as a percentage (0%–100%). PACU recovery quality was assessed using the Aldrete score, which ranges from 0 to 10, with higher scores indicating better recovery of consciousness and vital signs. The Aldrete score evaluates five domains: activity, respiration, circulation, consciousness, and oxygenation, each scored 0–2. Respiratory‐related adverse events within 48 h postoperatively included hypoxemia, respiratory depression, and upper airway obstruction. Hypoxemia was defined as SpO_2_ < 90% sustained for ≥ 30 s or requiring supplemental oxygen or airway intervention. Respiratory depression was defined as a respiratory rate < 8 breaths per minute or necessitating stimulation, airway management, or assisted ventilation by healthcare staff. Upper airway obstruction was defined as obvious airflow limitation, snoring‐like respiration, or requiring positional adjustment, airway maneuvers, or intubation. The initiation of CPAP or noninvasive positive pressure ventilation (NIPPV) during hospitalization was recorded to assess escalation of respiratory support.

#### 2.4.2. Postoperative Analgesia and Recovery Outcomes

To assess the effect of individualized nursing on analgesia and early functional recovery, retrospective data were collected on pain scores, perioperative opioid consumption, functional recovery time, postoperative adverse events, and patient comfort and sleep quality during hospitalization. Pain intensity was evaluated using the visual analog scale (VAS, 0–10), where 0 indicates *no pain* and 10 represents *the worst imaginable pain*, measured in the PACU, at 24 h, and at 48 h postoperatively. Perioperative opioid consumption was converted to morphine equivalent dose (MED) according to international equivalence standards. Time to first ambulation was recorded as the interval (in hours) from the end of surgery to the patient’s first standing or walking at the bedside. Postoperative adverse events—including postoperative nausea and vomiting (PONV), excessive sedation, and respiratory depression related to analgesia—were identified through nursing and medication records. Patient comfort and short‐term sleep quality during hospitalization were retrospectively assessed using the hospital nursing comfort scale (0–10, 0 = *extremely uncomfortable*, 10 = *extremely comfortable*) and the numerical rating scale (NRS) for sleep quality (0–10, 0 = *extremely poor sleep*, 10 = *excellent sleep*), respectively.

#### 2.4.3. Postoperative Sleep and Quality‐of‐Life Outcomes

Postoperative sleep and quality of life were assessed to evaluate the long‐term impact of individualized nursing. Data were retrospectively collected through outpatient follow‐up records, device‐exported data, and telephone interviews, including daytime sleepiness, improvement in nocturnal symptoms, CPAP adherence, quality of life, and respiratory‐related revisits. Daytime sleepiness was assessed using the Epworth Sleepiness Scale (ESS, 0–24), with higher scores indicating greater sleepiness. Improvement in nocturnal snoring and awakening episodes was documented based on subjective reports from patients or caregivers. CPAP adherence was evaluated via patient self‐report or device‐recorded usage, with average nightly use ≥ 4 h considered adherent. Quality of life was assessed using the 36‐Item Short Form Health Survey (SF‐36), which includes eight domains: physical functioning, role physical, bodily pain, general health, vitality, social functioning, role emotional, and mental health, scored 0–100, with higher scores indicating better quality of life. Respiratory‐related outpatient visits or emergency visits within 6 months postoperatively included any return visit due to dyspnea, hypoxemia, worsening OSA symptoms, or airway complications. Multiple visits by the same patient within the observation period were counted as a single event.

#### 2.4.4. Perioperative Safety and Healthcare Resource Utilization Outcomes

Perioperative safety and healthcare resource utilization outcomes included postoperative complications, intraoperative blood loss, postoperative drainage volume, ICU transfers, length of hospital stay, hospitalization costs, and readmissions. Postoperative complications were identified through medical records and discharge diagnoses and included pulmonary infection, respiratory failure, cardiovascular events, and serious adverse events requiring reintubation. Intraoperative blood loss was recorded as the actual blood loss during surgery. Postoperative drainage volume was recorded as the cumulative output from surgical drains. ICU transfer was defined as the transfer of patients from general wards to the intensive care unit due to respiratory or hemodynamic instability for continuous monitoring or respiratory support. Length of hospital stay was calculated in calendar days from admission to discharge. Total hospitalization costs included perioperative surgical, anesthesia, medication, nursing, and examination‐related expenses, as recorded in the hospital financial system. Postoperative readmission was defined as readmission within 30 or 90 days postoperatively due to surgery‐related or perioperative respiratory complications, with multiple readmissions within the same observation period counted as a single event.

### 2.5. Data Sources and Collection

This study retrospectively collected data from the electronic medical records, anesthesia, nursing, and hospital billing systems of patients with OSA. Extracted information included demographic characteristics (age, sex, and BMI), OSA‐related indices (PSG parameters, STOP‐Bang score, and prior CPAP use), perioperative treatments and nursing interventions, and key postoperative and follow‐up outcomes (oxygen saturation monitoring, respiratory events, pain scores, comfort, sleep quality, PONV, intraoperative blood loss and postoperative drainage, complications, length of hospital stay, hospitalization costs, number of revisits, ESS scores, CPAP adherence, and quality of life). All data were independently extracted and entered into a de‐identified database by two researchers, and any discrepancies were resolved by a third researcher.

### 2.6. Statistical Analysis

All statistical analyses were performed using SPSS Version 26.0 (IBM, Armonk, NY, USA). Continuous variables were assessed for normality before analysis. Normally distributed data are presented as mean ± standard deviation (*x̄* ± SD), while non‐normally distributed data are expressed as median (interquartile range) [*M* (P25, P75)]. Categorical variables are presented as counts and percentages (*n*, %). Between‐group comparisons of continuous variables with normal distribution and equal variance were performed using independent‐samples *t*‐tests, whereas non‐normally distributed variables were compared using the Mann–Whitney *U* test. Paired data, including pre‐ and post‐treatment or repeated follow‐up measurements, were analyzed using paired *t*‐tests or Wilcoxon signed‐rank tests. Categorical variables were compared using the chi‐square test or Fisher’s exact test as appropriate. Missing data were assessed before analysis. Variables with < 10–20% missing values were handled using multiple imputation, whereas cases with > 20% missing data were excluded from the analysis. All statistical tests were two‐sided, and a *p* value < 0.05 was considered statistically significant.

## 3. Results

### 3.1. Baseline Characteristics

**FIGURE 1:**
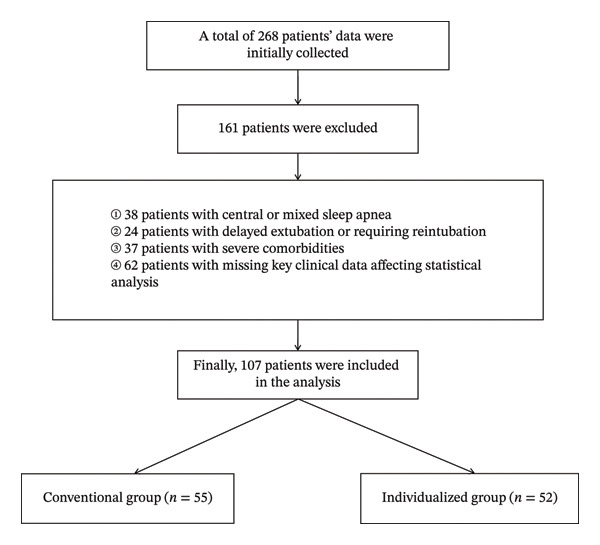
The flowchart of patient inclusion in this study.

A total of 107 patients with OSA were included, with 55 patients in the conventional group and 52 patients in the individualized group. The flowchart of patient inclusion is shown in Figure 1. No statistically significant differences were observed between the two groups in terms of age, sex, BMI, or ASA classification (all *p* > 0.05). The prevalence of comorbidities, including hypertension, diabetes, coronary artery disease, and chronic pulmonary disease, was similar between groups. Perioperative indicators, including AHI, preoperative minimum SpO_2_, nocturnal hypoxemia index, and STOP‐Bang score, also showed no significant differences. Prior CPAP use, surgical type, anesthesia method, duration of surgery, and perioperative analgesia strategies were comparably distributed between groups (Table 1). These balanced baseline characteristics provided a reliable foundation for the retrospective analysis of perioperative nursing interventions.

**TABLE 1:** Baseline characteristics of the study population.

Variable	Conventional group (*n* = 55)	Individualized group (*n* = 52)	*t*/*χ* ^2^	*p*
Age (years, mean ± SD)	58.7 ± 10.2	59.9 ± 9.8	−0.651	0.524
Sex, *n* (%)			0.007	0.935
Male	38 (69.1)	37 (71.2)		
Female	17 (30.9)	16 (30.8)		
BMI (kg/m^2^, mean ± SD)	30.1 ± 4.5	30.8 ± 4.2	−0.798	0.435
ASA classification, *n* (%)			0.092	0.955
I	5 (9.1)	4 (7.7)		
II	32 (58.2)	30 (57.7)		
III	18 (32.7)	18 (34.6)		
Hypertension, *n* (%)	28 (50.9)	30 (57.7)	0.495	0.482
Diabetes mellitus, *n* (%)	15 (27.3)	11 (21.2)	0.544	0.461
Coronary heart disease or cardiovascular disease, *n* (%)	12 (21.8)	11 (21.2)	0.007	0.933
Chronic pulmonary disease, *n* (%)	8 (14.5)	7 (13.5)	0.026	0.872
AHI (events/hour, mean ± SD)	34.5 ± 14.3	35.8 ± 15.1	−0.426	0.683
Lowest preoperative oxygen saturation (mean ± SD)	78.6 ± 8.9	77.9 ± 9.2	0.36	0.727
Nocturnal oxygen desaturation index (mean ± SD)	12.2 ± 6.5	12.8 ± 7.0	−0.449	0.668
Preoperative STOP‐Bang score (points, mean ± SD)	5.2 ± 1.1	5.3 ± 1.2	−0.453	0.651
Regular preoperative CPAP use, *n* (%)	10 (18.2)	14 (26.9)	1.174	0.279
Type of surgery, *n* (%)			0.301	0.960
Upper airway/head and neck	18 (32.7)	19 (36.5)		
Abdominal surgery	16 (29.1)	15 (28.8)		
Orthopedic surgery	13 (23.6)	12 (23.1)		
Other surgeries	8 (14.5)	6 (11.5)		
Anesthesia type, *n* (%)			0.375	0.541
General anesthesia	40 (72.7)	35 (67.3)		
Regional anesthesia	15 (27.3)	17 (32.7)		
Duration of surgery (min, mean ± SD)	115.4 ± 40.8	118.9 ± 42.1	−0.446	0.663
Perioperative analgesia strategy, *n* (%)			0.196	0.658
Multimodal analgesia	22 (40.0)	23 (44.2)		
Single‐modality analgesia	33 (60.0)	29 (55.8)		

*Note:* Data are presented as mean ± SD or number (%).

Abbreviations: AHI, apnea–hypopnea index; ASA, American Society of Anesthesiologists physical status classification; BMI, body mass index; CPAP, continuous positive airway pressure.

### 3.2. Comparison of Perioperative Respiratory Safety Outcomes

Perioperative respiratory safety outcomes differed between the two groups (Table 2). During PACU stay, the minimum SpO_2_ in the individualized group was 93.3 ± 3.8%, significantly higher than 90.4 ± 4.9% in the conventional group (*p* = 0.001). At PACU discharge, the Aldrete score was 9.1 ± 0.7 in the individualized group, compared with 8.4 ± 0.9 in the conventional group (*p* < 0.001). Within 48 h postoperatively, the incidence of hypoxemia, respiratory depression, and upper airway obstruction in the individualized group was 15.4%, 5.8%, and 7.7%, respectively, compared with 34.5%, 20.0%, and 21.8% in the conventional group. All differences were statistically significant (*p* < 0.05). The overall incidence of respiratory‐related adverse events was 21.2% in the individualized group, significantly lower than 45.5% in the standard care group (*p* = 0.008). Additionally, the proportion of patients requiring CPAP or noninvasive ventilation during hospitalization was lower in the individualized group compared with the conventional group (13.5% vs. 29.1%, *p* = 0.049).

**TABLE 2:** Comparison of perioperative respiratory safety outcomes between the two groups.

Variable	Conventional group (*n* = 55)	Individualized group (*n* = 52)	*t*/*χ* ^2^	*p*
Lowest SpO_2_ in PACU (%, mean ± SD)	90.4 ± 4.9	93.3 ± 3.8	−3.402	0.001
Aldrete score at PACU discharge (0–10, mean ± SD)	8.4 ± 0.9	9.1 ± 0.7	−4.287	< 0.001
Respiratory‐related adverse events within 48 h postoperatively, *n* (%)				
Hypoxemia events	19 (34.5)	8 (15.4)	5.201	0.023
Respiratory depression events	11 (20.0)	3 (5.8)	4.760	0.029
Upper airway obstruction events	12 (21.8)	4 (7.7)	4.194	0.041
Total respiratory‐related adverse events	25 (45.5)	11 (21.2)	7.070	0.008
Initiation of CPAP or noninvasive ventilation during hospitalization, *n* (%)	16 (29.1)	7 (13.5)	3.869	0.049

*Note:* Data are presented as mean ± SD or number (%). Respiratory‐related adverse events were defined as hypoxemia, respiratory depression, or upper airway obstruction occurring within 48 h postoperatively. *p* < 0.05 considered statistically significant.

Abbreviations: CPAP, continuous positive airway pressure; PACU, postanesthesia care unit; SpO_2_, peripheral oxygen saturation.

### 3.3. Comparison of Postoperative Analgesia and Functional Recovery

Postoperative analgesia and functional recovery outcomes differed between the two groups (Table 3). In the PACU and at 24 and 48 h postoperatively, VAS scores in the individualized group were 3.5 ± 1.0, 3.0 ± 0.9, and 2.4 ± 0.8, respectively, significantly lower than those in the conventional group (4.6 ± 1.2, 4.1 ± 1.1, and 3.2 ± 1.0; *p* < 0.001). Cumulative perioperative opioid consumption was 27.8 ± 9.6 mg in the individualized group, significantly lower than 38.6 ± 12.4 mg in the conventional group (*p* < 0.001). Furthermore, patients in the individualized group had an earlier time to first ambulation, higher postoperative sleep quality scores, and greater comfort during hospitalization compared with the conventional group (all *p* < 0.001). Regarding adverse events, the incidence of PONV and analgesia‐related complications was lower in the individualized group (*p* < 0.05).

**TABLE 3:** Comparison of postoperative analgesia and functional recovery outcomes between the two groups.

Variable	Conventional group (*n* = 55)	Individualized group (*n* = 52)	*t*/*χ* ^2^	*p*
VAS pain score (0–10, mean ± SD)				
PACU	4.6 ± 1.2	3.5 ± 1.0	5.136	< 0.001
24 h postoperatively	4.1 ± 1.1	3.0 ± 0.9	5.508	< 0.001
48 h postoperatively	3.2 ± 1.0	2.4 ± 0.8	4.407	< 0.001
Perioperative opioid consumption (morphine equivalents, mg, mean ± SD)	38.6 ± 12.4	27.8 ± 9.6	5.017	< 0.001
Time to first ambulation (h, mean ± SD)	30.5 ± 8.6	22.9 ± 6.7	5.028	< 0.001
Postoperative sleep quality score (NRS, 0–10, mean ± SD)	6.1 ± 1.3	7.4 ± 1.2	−5.246	< 0.001
PONV, *n* (%)	19 (36.5)	9 (17.3)	4.111	0.043
Analgesia‐related adverse events, *n* (%)	14 (26.9)	5 (9.6)	4.592	0.032
In‐hospital comfort score (0–10, mean ± SD)	6.5 ± 1.2	7.9 ± 1.0	−6.437	< 0.001

*Note:* Opioid consumption was converted to morphine equivalents. *p* < 0.05 considered statistically significant.

Abbreviations: NRS, numerical rating scale; PONV, postoperative nausea and vomiting; VAS, visual analog scale.

### 3.4. Comparison of Postoperative Symptom Improvement and Quality of Life

Postoperative daytime sleepiness, nocturnal symptoms, and CPAP adherence differed between the two groups (Table 4). ESS scores in the individualized group at 1, 3, and 6 months postoperatively were 9.4 ± 2.8, 7.8 ± 2.6, and 6.9 ± 2.3, respectively, compared with 11.8 ± 3.2, 10.5 ± 3.0, and 9.6 ± 2.8 in the conventional group (*p* < 0.001). The proportion of patients reporting improvement in nocturnal snoring and awakening episodes, as well as CPAP adherence, was higher in the individualized group. Average nightly CPAP use was 5.8 ± 1.3 h in the individualized group versus 4.1 ± 1.5 h in the conventional group (*p* < 0.001). Although respiratory‐related revisits or readmissions tended to be lower in the individualized group, the differences did not reach statistical significance (*p* > 0.05). Quality‐of‐life assessment using the SF‐36 showed that all domains—physical functioning, role physical, bodily pain, general health, vitality, social functioning, role emotional, and mental health—were significantly higher in the individualized group at 1, 3, and 6 months postoperatively (*p* < 0.001; Table 5).

**TABLE 4:** Comparison of postoperative daytime sleepiness, nocturnal symptom improvement, and CPAP adherence between the two groups.

Variable	Conventional group (*n* = 55)	Individualized group (*n* = 52)	*t*/*χ* ^2^	*p*
ESS daytime sleepiness score (0–24, mean ± SD)				
Month 1	11.8 ± 3.2	9.4 ± 2.8	4.117	< 0.001
Month 3	10.5 ± 3.0	7.8 ± 2.6	5.106	< 0.001
Month 6	9.6 ± 2.8	6.9 ± 2.3	5.492	< 0.001
Improvement in nocturnal snoring and nocturnal choking awakenings, *n* (%)				
Month 1	29 (52.7)	38 (73.1)	4.729	0.030
Month 3	32 (58.2)	42 (80.8)	6.393	0.011
Month 6	34 (61.8)	45 (86.5)	8.454	0.004
CPAP adherence rate, *n* (%)				
Month 1	22 (40.0)	33 (63.5)	5.890	0.015
Month 3	25 (45.5)	37 (71.2)	7.244	0.007
Month 6	26 (47.3)	40 (76.9)	9.942	0.002
Average daily CPAP usage time (h/night, mean ± SD)	4.1 ± 1.5	5.8 ± 1.3	−6.178	< 0.001
Respiratory‐related revisits/readmissions, *n* (%)				
Month 1	10 (18.2)	4 (7.7)	2.586	0.108
Month 3	12 (21.8)	5 (9.6)	2.978	0.084
Month 6	14 (25.5)	6 (11.5)	3.406	0.065

*Note:* CPAP adherence was defined as an average daily usage time ≥ 4 h. *p* < 0.05 considered statistically significant.

Abbreviation: ESS, Epworth Sleepiness Scale (range 0–24).

**TABLE 5:** Comparison of postoperative SF‐36 quality‐of‐life outcomes between the two groups.

Variable	Conventional group (*n* = 55)	Individualized group (*n* = 52)	*t*	*p*
Physical functioning (0–100)				
Month 1	68.4 ± 10.2	75.6 ± 9.1	−3.872	< 0.001
Month 3	72.1 ± 9.8	81.2 ± 8.6	−5.007	< 0.001
Month 6	75.6 ± 9.3	85.4 ± 8.2	−5.773	< 0.001
Role physical (0–100)				
Month 1	62.3 ± 12.4	70.8 ± 11.2	−3.668	< 0.001
Month 3	67.9 ± 11.8	77.6 ± 10.1	−4.737	< 0.001
Month 6	71.8 ± 11.1	82.3 ± 9.5	−5.284	< 0.001
Bodily pain (0–100)				
Month 1	65.1 ± 9.6	72.3 ± 8.8	−4.048	< 0.001
Month 3	69.2 ± 9.3	78.5 ± 8.1	−5.464	< 0.001
Month 6	72.6 ± 8.8	82.1 ± 7.6	−6.011	< 0.001
General health (0–100)				
Month 1	63.4 ± 8.9	70.5 ± 8.0	−4.362	< 0.001
Month 3	67.8 ± 8.4	76.4 ± 7.3	−5.604	< 0.001
Month 6	71.2 ± 8.1	80.1 ± 6.9	−6.028	< 0.001
Vitality (0–100)				
Month 1	60.5 ± 9.1	68.8 ± 8.2	−5.027	< 0.001
Month 3	65.2 ± 8.6	75.4 ± 7.5	−6.365	< 0.001
Month 6	69.4 ± 8.0	80.2 ± 6.9	−7.445	< 0.001
Social functioning (0–100)				
Month 1	66.7 ± 10.3	74.6 ± 9.2	−4.159	< 0.001
Month 3	70.5 ± 9.8	80.1 ± 8.5	−5.352	< 0.001
Month 6	74.1 ± 9.2	84.6 ± 7.8	−6.457	< 0.001
Role emotional (0–100)				
Month 1	64.8 ± 11.5	72.9 ± 10.3	−3.794	< 0.001
Month 3	69.1 ± 10.8	78.8 ± 9.2	−5.078/	< 0.001
Month 6	73.5 ± 10.1	84.2 ± 8.7	−6.045	< 0.001
Mental health (0–100)				
Month 1	67.2 ± 9.4	74.8 ± 8.3	−4.404	< 0.001
Month 3	71.6 ± 8.9	80.2 ± 7.6	−5.536	< 0.001
Month 6	75.3 ± 8.4	85.1 ± 7.1	−6.538	< 0.001

*Note:* All domain scores range from 0 to 100, with higher scores indicating better health‐related quality of life. *p* < 0.05 considered statistically significant.

Abbreviation: SF‐36, 36‐Item Short Form Health Survey.

### 3.5. Comparison of Perioperative Safety and Healthcare Resource Utilization

Perioperative safety and healthcare resource utilization outcomes differed between the two groups (Table 6). The incidence of postoperative respiratory failure was 1.9% in the individualized group, significantly lower than 12.7% in the conventional group (*p* = 0.034). The rates of pulmonary infection, cardiovascular events, and reintubation did not differ significantly between groups. The overall incidence of postoperative complications was 13.5% in the individualized group, compared with 32.7% in the conventional group (*p* = 0.019). ICU transfer rates were 7.7% and 21.8% in the individualized and conventional groups, respectively (*p* = 0.041). Intraoperative blood loss and postoperative drainage volume did not differ significantly between the groups. Length of hospital stay and total hospitalization costs were lower in the individualized group, with a mean stay of 7.8 ± 2.1 days and costs of 57,263 ± 13,487 CNY, compared with 9.6 ± 2.8 days and 68,540 ± 15,322 CNY in the conventional group (*p* < 0.001).

**TABLE 6:** Comparison of perioperative safety and healthcare resource utilization outcomes between the two groups.

Variable	Conventional group (*n* = 55)	Individualized group (*n* = 52)	*t*/*χ* ^2^	*p*
Postoperative complications, *n* (%)				
Pulmonary infection	9 (16.4)	3 (5.8)	3.013	0.083
Respiratory failure	7 (12.7)	1 (1.9)	4.510	0.034
Cardiovascular events	7 (12.7)	3 (5.8)	1.527	0.217
Reintubation	5 (9.1)	1 (1.9)	2.594	0.107
Total postoperative complications, *n* (%)	18 (32.7)	7 (13.5)	5.541	0.019
Intraoperative blood loss (mL, mean ± SD)	245.6 ± 118.4	231.8 ± 110.2	0.641	0.520
Postoperative drainage volume (mL, mean ± SD)	312.5 ± 135.6	268.4 ± 120.9	1.774	0.079
ICU admission, *n* (%)	12 (21.8)	4 (7.7)	4.194	0.041
Length of hospital stay (days, mean ± SD)	9.6 ± 2.8	7.8 ± 2.1	3.871	< 0.001
Total hospitalization cost (CNY, mean ± SD)	68,540 ± 15,322	57,263 ± 13,487	4.075	< 0.001

*Note:* Total postoperative complications refer to the occurrence of any complication listed in this table during hospitalization. *p* < 0.05 considered statistically significant.

Abbreviations: CNY, Chinese yuan; ICU, intensive care unit; SD, standard deviation.

## 4. Discussion

OSA is a common yet frequently underdiagnosed condition worldwide, associated with a markedly increased risk of cardiopulmonary complications during the perioperative period, including respiratory failure, hypertension, arrhythmias, and unexpected mortality [14, 18, 19]. Clinical nursing models are shifting from standardized protocols toward individualized approaches, which integrate preoperative screening, risk stratification, and perioperative adjustments to better accommodate patient‐specific risk profiles [13]. In this retrospective cohort study, patients in the individualized group demonstrated superior outcomes compared with the conventional group across multiple domains, including respiratory safety, analgesia, sleep and quality of life, complication rates, ICU transfers, and hospital resource utilization.

Patients with OSA are particularly vulnerable to perioperative hypoxemia, respiratory depression, and upper airway obstruction, which are closely linked to an increased risk of postoperative complications, prolonged hospitalization, and adverse prognoses, thereby imposing a substantial burden on patients [20]. A systematic review by Titu et al. [21] highlighted that OSA patients are at elevated risk of perioperative respiratory depression, hypoxemia, and related complications. Individualized care pathways—including CPAP therapy, stratified risk management, and multimodal analgesia—have been shown to effectively reduce these risks. However, outcomes, such as Aldrete scores and the incidence of adverse events, vary depending on surgical type and patient adherence, suggesting that nursing models, risk assessment tools, and patient characteristics may all influence final outcomes. Consistent with these findings, our study demonstrated that the individualized group achieved higher minimum SpO_2_ in the PACU, higher Aldrete scores, and lower rates of respiratory adverse events within 48 h postoperatively, as well as reduced need for respiratory support during hospitalization, compared with the conventional group. These results reinforce the importance of optimized perioperative management in enhancing respiratory safety for OSA patients. Furthermore, evidence suggests that coordinated, multidimensional interventions—including systematic risk stratification, ensuring preoperative CPAP adherence, intraoperative positioning, and continuous postoperative respiratory monitoring—can collectively reduce the incidence of respiratory complications. Strategies, such as graded screening, enhanced postoperative monitoring, and optimization of patient care pathways, are supported by empirical studies and are closely aligned with the interventions applied in this study [22, 23]. These findings underscore the clinical significance of implementing individualized, full‐process perioperative respiratory safety management for surgical patients with OSA, highlighting its potential to improve outcomes and reduce perioperative risks.

OSA patients are particularly sensitive to opioids and are prone to postoperative respiratory depression, making the balance between analgesia and respiratory safety a central focus of perioperative management [24]. In this study, the individualized group demonstrated superior outcomes compared with the conventional group in terms of postoperative pain control, functional recovery, sleep quality, and patient comfort. Similarly, previous studies have shown that multimodal analgesia combined with comprehensive nursing can reduce opioid consumption, alleviate pain, decrease respiratory complications, and enhance functional recovery, although specific interventions and outcomes vary depending on surgical type and patient characteristics [25]. Moreover, multimodal analgesia, individualized pain management strategies, early mobilization, and health education act synergistically in OSA patients to reduce opioid‐related respiratory depression, optimize sleep, and accelerate recovery [18]. Despite limitations in existing evidence due to disease heterogeneity and study design, perioperative management is increasingly moving toward multidimensional integration and pathway optimization. For high‐risk OSA patients classified as ASA III–IV, analgesia management and enhanced recovery pathways require rigorous methodology; coordinated, individualized nursing and anesthetic interventions may improve clinical outcomes and provide a practical foundation for innovation in perioperative care.

Long‐term postoperative outcomes in OSA patients are often affected by persistent daytime sleepiness, frequent nocturnal respiratory events, and suboptimal CPAP adherence, which in turn impact quality of life and recovery [26]. Our follow‐up data demonstrated that individualized nursing was associated with improvements in ESS scores, CPAP adherence, and quality of life, accompanied by reductions in nocturnal symptom burden and respiratory‐related revisits. Optimization of CPAP adherence and quality of life is influenced by multiple interventions, including health education, behavioral motivation, remote follow‐up, and stratified management [27–29]. For instance, a systematic review by Labarca et al. indicated that telemedicine and structured follow‐up can increase CPAP usage duration, while Rapelli et al. highlighted that motivational interventions are more effective than standard care in improving adherence, although their sustainability and mechanisms remain under debate [28, 29]. This study innovatively integrated health education, staged follow‐up, and adherence management into an individualized nursing pathway, reinforcing multidimensional mechanism integration. This approach demonstrated advantages in sustained outcome improvement, control of revisits, and standardization of nursing processes. Implementation of coordinated interventions may facilitate the development of structured postoperative follow‐up care for OSA patients and provide a theoretical foundation and potential optimization framework for future multicenter cohort studies and mechanistic research.

Patients with OSA face heightened perioperative risks, including severe complications, increased ICU transfers, and elevated hospitalization costs, a dual burden on safety and healthcare resources that has been widely documented in both international and domestic studies [30]. Consequently, effectively mitigating complication risks while managing high resource utilization has become a critical focus of clinical perioperative management. In this study, the individualized group demonstrated significant advantages over the conventional group in key outcomes, including incidence of severe complications, ICU transfer rate, length of hospital stay, and associated hospitalization costs, reflecting dual benefits in patient safety and resource utilization. Dawson et al. highlighted that OSA patients often encounter economic and management pressures, such as surgical delays and insufficient monitored beds, due to increased postoperative complications and ICU resource demands [31]. Our findings suggest that individualized nursing can achieve tangible resource savings and clinical efficacy, offering a novel pathway for management innovation. Accordingly, multidisciplinary team collaboration and process redesign to optimize resource utilization are pivotal for achieving both safety and economic benefits. These results provide robust evidence and practical guidance for enhancing perioperative safety, optimizing resource allocation, and advancing nursing management systems.

However, this study has several limitations. First, this study was a single‐center retrospective cohort, with nursing group allocation based on prior clinical records, which may introduce selection bias and potential confounding. Second, the sample size was relatively limited, and certain outcomes, such as revisit rates, may lack sufficient statistical power. Third, some follow‐up outcomes relied on patient self‐report and nursing documentation, introducing potential information bias. Fourth, patients with severe comorbidities were excluded to reduce clinical heterogeneity and confounding, which may limit the generalizability of the findings to medically complex OSA patients. Finally, follow‐up was limited to 6 months, primarily reflecting short‐ to mid‐term outcomes, and long‐term efficacy remains to be established. Future research should focus on multicenter prospective randomized controlled trials, with expanded sample sizes, extended follow‐up periods, and standardization of individualized nursing pathways. In addition, studies specifically targeting OSA patients with severe comorbidities are warranted to evaluate subgroup‐specific effects and to determine the effectiveness and safety of individualized nursing in this population, thereby enhancing external validity and clinical applicability of findings.

## 5. Conclusion

In this retrospective cohort study, individualized nursing was significantly associated with improved perioperative respiratory safety, reduced analgesic requirements, accelerated early functional recovery, enhanced long‐term sleep and quality of life, and decreased incidence of complications and healthcare resource consumption in patients with OSA. These findings suggest that integrating stratified assessment and multidimensional interventions into conventional care may optimize perioperative outcomes for OSA patients. However, causal relationships require further verification through multicenter prospective studies.

## Author Contributions

Qunyu Lin is responsible for the study concepts and design, definition of intellectual content, literature research, and manuscript preparation and editing. Yihong Yang is responsible for the definition of intellectual content, literature research, clinical studies, data acquisition and analysis, and statistical analysis. Chunmiao Huang and Dongdong Huang are responsible for the clinical studies, data acquisition and analysis, and statistical analysis. Chao Fang is responsible for the guarantor of the integrity of the entire study and manuscript review.

## Funding

The authors have nothing to report.

## Disclosure

All authors read and approved the final manuscript.

## Ethics Statement

The study was approved by the Ethics Committee of the First Hospital of Putian (Approval No. 2026‐009). Due to the retrospective design, the requirement for informed consent was waived.

## Consent

Please see the Ethics Statement.

## Conflicts of Interest

The authors declare no conflicts of interest.

## Data Availability

The experimental data used to support the findings of this study are available from the corresponding author upon request.

## References

[1] Zaki I. , Phyu S. L. , and Turnbull C. , Obstructive Sleep Apnoea, Medicine. (2023) 51, no. 11, 806–812, 10.1016/j.mpmed.2023.08.004.

[2] Ooi E. L. and Rajendran S. , Obstructive Sleep Apnea in Coronary Artery Disease, Current Problems in Cardiology. (2023) 48, no. 8, 10.1016/j.cpcardiol.2022.101178. 35341799

[3] Keenan B. , de Chazal P. , Penzel T. et al., 0431 Different Physiological Characteristics of Obstructive Sleep Apnea Symptom Subtypes Across International Sleep Centers, Sleep. (2023) 46, no. Supplement_1, A191–A192, 10.1093/sleep/zsad077.0431.

[4] Frangopoulos F. , Nicolaou I. , Zannetos S. et al., Estimating Obstructive Sleep Apnea in Cyprus: A Randomised, Stratified Epidemiological Study Using STOP-Bang Sleep Apnea Questionnaire, Sleep Medicine. (2019) 61, 37–43, 10.1016/j.sleep.2019.04.013. 31285161

[5] Bouchech R. , Jmeaa M. B. , Baklouti M. et al., Update on the Epidemiology of Obstructive Sleep Apnea Syndrome in Central Eastern Tunisia Over 11 Years, Pan African Medical Journal. (2024) 47, 10.11604/pamj.2024.47.206.42949. PMC11380607 39247780

[6] Wali S. O. , Abalkhail B. , and Krayem A. , Prevalence and Risk Factors of Obstructive Sleep Apnea Syndrome in a Saudi Arabian Population, Annals of Thoracic Medicine. (2017) 12, no. 2, 88–94, 10.4103/1817-1737.203746. 28469718 PMC5399696

[7] Sunwoo J.-S. , Hwangbo Y. , Kim W.-J. , Chu M. K. , Yun C.-H. , and Yang K. I. , Prevalence, Sleep Characteristics, and Comorbidities in a Population at High Risk for Obstructive Sleep Apnea: A Nationwide Questionnaire Study in South Korea, PLoS One. (2018) 13, no. 2, 10.1371/journal.pone.0193549. PMC5831105 29489913

[8] Aurora R. N. and Quan S. F. , Quality Measure for Screening for Adult Obstructive Sleep Apnea by Primary Care Physicians, Journal of Clinical Sleep Medicine. (2016) 12, no. 8, 1185–1187, 10.5664/jcsm.6064. 27397659 PMC4957197

[9] Faria A. , Allen A. H. , Fox N. , Ayas N. , and Laher I. , The Public Health Burden of Obstructive Sleep Apnea, Sleep Science. (2021) 14, no. 3, 257–265, 10.5935/1984-0063.20200111. 35186204 PMC8848533

[10] Jiahuan X. , Ying Z. , Hongyu J. et al., Serum sTREM2: A Potential Biomarker for Mild Cognitive Impairment in Patients With Obstructive Sleep Apnea, Frontiers in Aging Neuroscience. (2022) 14, 10.3389/fnagi.2022.843828. PMC9125145 35615588

[11] Borsoi L. , Armeni P. , Donin G. , Costa F. , and Ferini-Strambi L. , The Invisible Costs of Obstructive Sleep Apnea (OSA): Systematic Review and Cost-of-Illness Analysis, PLoS One. (2022) 17, no. 5, 10.1371/journal.pone.0268677. PMC9122203 35594257

[12] Seet E. , Nagappa M. , and Wong D. T. , Airway Management in Surgical Patients With Obstructive Sleep Apnea, Anesthesia & Analgesia. (2021) 132, no. 5, 1321–1327, 10.1213/ANE.0000000000005298. 33857974

[13] Chaudhry R. A. , Zarmer L. , West K. , and Chung F. , Obstructive Sleep Apnea and Risk of Postoperative Complications After Non-Cardiac Surgery, Journal of Clinical Medicine. (2024) 13, no. 9, 10.3390/jcm13092538. PMC11084150 38731067

[14] Sleep Respiratory Disorders Working Committee of the Respiratory Medicine Branch of the Chinese Medical Doctor Association , Chinese Society of Anesthesiology of Chinese Medical Association , and National Clinical Research Center for Geriatric Diseases , Expert Consensus on Perioperative Management of Adult Patients Combined With Obstructive Sleep Apnea (2025 Edition), Zhonghua Yixue Zazhi. (2025) 105, no. 14, 1045–1054, 10.3760/cma.j.cn112137-20240814-01874. 40176650

[15] Duong-Quy S. , Nguyen-Huu H. , Hoang-Chau-Bao D. et al., Personalized Medicine and Obstructive Sleep Apnea, Journal of Personalized Medicine. (2022) 12, no. 12, 10.3390/jpm12122034. PMC9781564 36556255

[16] Hilbert J. and Yaggi H. K. , Patient-Centered Care in Obstructive Sleep Apnea: A Vision for the Future, Sleep Medicine Reviews. (2018) 37, 138–147, 10.1016/j.smrv.2017.02.004. 28633915 PMC6006997

[17] Sutherland K. , Yee B. J. , Kairaitis K. , Wheatley J. , de Chazal P. , and Cistulli P. A. , A Phenotypic Approach for Personalised Management of Obstructive Sleep Apnoea, Current Otorhinolaryngology Reports. (2021) 9, no. 3, 223–237, 10.1007/s40136-021-00346-6.

[18] Cozowicz C. and Memtsoudis S. G. , Perioperative Management of the Patient With Obstructive Sleep Apnea: A Narrative Review, Anesthesia & Analgesia. (2021) 132, no. 5, 1231–1243, 10.1213/ANE.0000000000005444. 33857965

[19] Yeghiazarians Y. , Jneid H. , Tietjens J. R. et al., Obstructive Sleep Apnea and Cardiovascular Disease: A Scientific Statement From the American Heart Association, Circulation. (2021) 144, no. 3, e56–e67, 10.1161/CIR.0000000000000988. 34148375

[20] Weingarten T. N. and Sprung J. , Perioperative Considerations for Adult Patients With Obstructive Sleep Apnea, Current Opinion in Anaesthesiology. (2022) 35, no. 3, 392–400, 10.1097/ACO.0000000000001125. 35671031

[21] Titu I. M. , Vulturar D. M. , Chis A. F. , Oprea A. , Manea A. , and Todea D. A. , Impact of Obstructive Sleep Apnea in Surgical Patients: A Systematic Review, Journal of Clinical Medicine. (2025) 14, no. 14, 10.3390/jcm14145095. PMC12295387 40725786

[22] Pappu A. and Singh M. , Best Perioperative Practices in the Management of Obstructive Sleep Apnea Patients Undergoing Ambulatory Surgery, Current Opinion in Anaesthesiology. (2024) 37, no. 6, 644–650, 10.1097/ACO.0000000000001441. 39476386

[23] Kirkin D. J. , Sabharwal A. , and Cousins J. , Obstructive Sleep Apnoea and Anaesthesia, Anaesthesia and Intensive Care Medicine. (2025) 26, no. 12, 824–832, 10.1016/j.mpaic.2025.10.014.

[24] Cozowicz C. , Chung F. , Doufas A. G. , Nagappa M. , and Memtsoudis S. G. , Opioids for Acute Pain Management in Patients with Obstructive Sleep Apnea: A Systematic Review, Anesthesia & Analgesia. (2018) 127, no. 4, 988–1001, 10.1213/ANE.0000000000003549. 29958218

[25] Cozowicz C. , Poeran J. , Zubizarreta N. et al., Non-Opioid Analgesic Modes of Pain Management Are Associated With Reduced Postoperative Complications and Resource Utilisation: A Retrospective Study of Obstructive Sleep Apnoea Patients Undergoing Elective Joint Arthroplasty, British Journal of Anaesthesia. (2019) 122, no. 1, 131–140, 10.1016/j.bja.2018.08.027. 30579391

[26] Lo Bue A. , Salvaggio A. , Iacono Isidoro S. , Romano S. , and Insalaco G. , OSA and CPAP Therapy: Effect of Gender, Somnolence, and Treatment Adherence on Health-Related Quality of Life, Sleep and Breathing. (2020) 24, no. 2, 533–540, 10.1007/s11325-019-01895-3. 31309464

[27] Mehrtash M. , Bakker J. P. , and Ayas N. , Predictors of Continuous Positive Airway Pressure Adherence in Patients With Obstructive Sleep Apnea, Lung. (2019) 197, no. 2, 115–121, 10.1007/s00408-018-00193-1. 30617618

[28] Labarca G. , Schmidt A. , Dreyse J. , Jorquera J. , and Barbe F. , Telemedicine Interventions for CPAP Adherence in Obstructive Sleep Apnea Patients: Systematic Review and Meta-Analysis, Sleep Medicine Reviews. (2021) 60, 10.1016/j.smrv.2021.101543. 34537668

[29] Rapelli G. , Pietrabissa G. , Manzoni G. M. et al., Improving CPAP Adherence in Adults With Obstructive Sleep Apnea Syndrome: A Scoping Review of Motivational Interventions, Frontiers in Psychology. (2021) 12, 10.3389/fpsyg.2021.705364. PMC8406627 34475840

[30] Sun X. , Yu J. , Luo J. , Xu S. , Yang N. , and Wang Y. , Meta-Analysis of the Association Between Obstructive Sleep Apnea and Postoperative Complications, Sleep Medicine. (2022) 91, 1–11, 10.1016/j.sleep.2021.11.019. 35231774

[31] Dawson D. , Singh M. , and Chung F. , The Importance of Obstructive Sleep Apnoea Management in Peri‐Operative Medicine, Anaethesia. (2016) 71, no. 3, 251–256, 10.1111/anae.13362. 26763386

